# Supplementary material for: Health knowledge and care seeking behaviour in resource-limited settings amidst the COVID-19 pandemic: A qualitative study in Ghana
Source: PLoS One. 2021 May 5;16(5):e0250940. doi: 10.1371/journal.pone.0250940 (PMC8099062; doi:10.1371/journal.pone.0250940)
Supplement: Attachment — Submitted filename: Response to Reviewers.docx [file pone.0250940.s001.docx]

**Response to Reviewers**

**Health Knowledge and Care Seeking Behaviour in Resource-Limited Settings amidst the COVID-19 Pandemic: A qualitative study in Ghana**

**Reviewer #1**

This manuscript presents novel results on the effect of COVID-19 on health knowledge and care seeking behaviours in Cape Coast Metropolis. However, there are few minor issues that need to be addressed to further improve the quality of the manuscript.

**Introduction**

Comment: The authors should consider revising the first sentence of the second paragraph that starts with “The increasing burden of COVID-19 has resulted in various international………………..”

**Response:** The sentence has been revised as suggested (See page 4).

Comment: The first sentence of the third paragraph needs a citation. This sentence reads “The emergence of COVID–19 has deepened the strain on health systems across the globe more especially the already overburdened……………….

**Response:** A citation has been provided as suggested by the reviewer (See page 4).

**Methods**

**Comment:** The statement that reads “The study followed the Consolidated Criteria for Reporting Qualitative Research (COREQ) guideline in reporting this study” needs to be cited.

**Response:** The statement has been cited (citation 27) (See page 9).

**Comment:** At the study population and sampling, could the authors specify the age category for the adult they used in their study? Also, could the authors justify why they included only residents who lived for at least 6months in the study?

**Response:** The study population involved adults aged 18 or older (see page 9). Also, the 6 months period begins from March, 2020 when Cape Coast Metropolis recorded first case of COVID following Ghana first recorded COVID-19 cases in March (see page 10).

**References**

**Comment:** The authors should work on the reference list. The numbers that need revision include 4, 12, 13, 15, 18, 20, 29, 34, 36, 46, and 48.

**Response:** Reference list has been revised and any errors corrected (See page 24).

**Reviewer #2**

Review of “Health Knowledge and Care Seeking Behaviour in Resource-Limited Settings amidst the COVID-19 Pandemic: A qualitative study in Ghana”. Generally, the entire manuscript requires some amount of editing, some of the phrases are poorly constructed, some of the sentences lack verbs, wrong phrases, wrong tenses, lack of parallel structure in a few of the sentences.

**Ethics statement**

**Comment:** Metropolitan Health Directorate IRB (DHRCIRB/15/05/17): Is it the Dodowa Health and Research Centre IRB or the Metropolitan health directorate that approved the study? Correct this.

**Response:** Approval was provided by the Cape Coast Metropolitan Health Directorate. This has thus been revised in the manuscript to remove the Dodowa Health and Research Centre which was an oversight (see page 11).

**Abstract**

Comment: Page 3: “COVID–19 associated conscious and unconscious reforms should systematically be harnessed”, need explanation or listing of exactly what authors are referring to.

**Response:** This has been explained in the concluding section of the manuscript. We felt that explaining in the abstract will cause it to exceed the expected number of words.

**Introduction**

Comment: Page 5, second sentence in the last paragraph is missing “of”

**Response:** “of” has been inserted in the second sentence of the last paragraph on page 5.

**Conceptual framework**

Comment; Paragraph 1: What are the three tenets? Mention them

**Response:** The three tenets have been stated (See page 6).

What are the few flaws of the model? List them and how this current study dealt or avoided them.

**Response:** This has been addressed (See page 7-8).

Comment: What are these tenets and how are they in line with the study objectives, kindly explain

**Response:** This has been addressed on page 6 and 8 of the manuscript.

Comment: Paragraph 1, the statement “This fit well with the study’s objective of assessing impact of the COVID-19 pandemic on the health knowledge of individuals.” Is that the entire objective of the study, I thought the model is more encompassing than making provision for only knowledge and the same with the study.

**Response:** This statement has been revised on page 6–7.

**Methods and materials**

Comment: Last sentence on page 8 to first sentence on page 9: Check the sentence, the tense is not appropriate.

**Response:** The sentence has been revised to correct the tense (See page 9)

**Study population and sampling**

Comment: Page 9, the following sentence “Only residents who had lived in the metropolis for at least six months within the period of COVID–19 pandemic in Ghana.” Says nothing, it is missing a verb. Check other paragraphs for such kinds of phrases as there are several in the entire manuscript.

**Response:** This sentence has been revised on page 10.

Comment: Page 9, kindly explain the following phrase “Recruitment was purposive and prospective.”

**Response:** The phrase “recruitment was purposive and prospective” has been revised on page 10

Comment: How was saturation attained? Kindly explain.

**Response:** This has been explained on page 10 of the revised manuscript

**Procedures**

Comment: Kindly complete title “procedure” does not mean anything.

**Response:** The title “procedures” has been clarified to “Study procedures” on page 10.

Comment: Consider improving the second sentence in the first paragraph, it is difficult to comprehend.

**Response:** The sentence has been revised to improve comprehension on page 10.

Comment: Kindly explain what this means “the instrument was self-developed from literature”

**Response:** the statement has been revised on page 10.

Comment: Please provide the following: age range of participants, any refusals, language that the study was carried in and how the research assistants were trained. Also, indicate how many other researchers were involved.

**Response:** The age range of the study participants (see page 10), languages used in the interviews and how the research assistants were trained, and other researchers involved in the data collection (page 11) have been included

Comment: Check the last sentence, I am not sure that “Collaborate” is the appropriate word, probably “corroborate”

**Response:** The sentence has been revised and “collaborate” replaced with “corroborate” on page 11.

**Ethical issues**

Comment: I am not sure the study “took” approval from the Metropolitan Health Directorate following ethical approval (DHRCIRB/15/05/17) from the Dodowa Research Centre. Correction: I think the study sought permission from the Metropolitan Health Directorate. Also, which Metropolitan Health Directorate are researchers referring to, kindly be specific. Also, correct the full name of the Dodowa Research Centre, it is not complete. Also correct it in the ethics statement and replace it with the Metropolitan Health Directorate.

**Response:** The ethical statement has been revised to remove Dodowa Health and Research Centre which was an oversight. The ethical approval was given by the Cape Coast Metropolitan Health Directorate which has been indicated on page 11.

**Data analysis**

Comment: I don't understand how literature review is reported as a theme derived from your analysis

**Response:** The statement sought to explain that some of the themes identified from the data were based on literature. It has thus, been revised for clarity on page 12.

Comment: Kindly explain “internal homogeneity” and “external heterogeneity”.

**Response:** Internal homogeneity and external heterogeneity have been explained in parenthesis on page 12.

**Results**

Comment: Pages 13-19: The results section as it stands needs some improvement in analysis and write up. Researchers have resorted to putting most of the information in direct quotations, without critically analyzing the crucial messages that they contain. For instance, several extensive quotes with very little explanation have been presented under all the themes. Authors should revise the results section by providing a deeper level of analysis with fewer quotes but more paraphrased and meaningful presentation.

**Response: The results section has been improved taken into consideration the concerns raised (see pages 13-21)**

Comment: Opening sentence in results section: mention only main themes and link them to the framework and delete other irrelevant information that does not add anything to the introduction.

**Response:** This section has been revised as suggested.

Comment: Table 1. Structure the third major theme in the table into negative and positive effects and list the sub themes of each under is rightful theme

**Response:** The table has been restructured as suggested on page 13.

Comment: Page 13, kindly explain or list “community public address systems”

**Response:** This has been revised to a more self-explanatory term “community information centres” on page 14.

Comment: Delete “the” from sentence with the following phrase “due to their availability to majority of the Ghana’s population”

**Response:** “the” has been deleted from the sentence on page 14.

**Discussion**

Comment: The authors do not refer to the conceptual framework in this section and the extent to which it helped them to answer their question or to achieve their study object. They also failed to indicate how they dealt with the flaws that were mentioned in the conceptual framework.

**Response:** The conceptual framework has been referred to throughout the discussion pointing out how it explains the observations made (see pages 23–22).

**Reviewer #3**

Comment: A couple of typographical errors and the use of articles have been noted and comments have been made in the attached document. The author(s) should be consistent in the language (Bristish or American English) choice.

**Response:** The manuscript has been revised to conform to British English and all typographical errors have been rectified.
